# Relationship between team climate and satisfaction at work in the Family Health Strategy

**DOI:** 10.11606/s1518-8787.2021055003307

**Published:** 2021-12-16

**Authors:** Marina Peduzzi, Heloise Lima Fernandes Agreli, Pilar Espinoza, Mitti Ayako Hara Koyama, Everson Meireles, Patrícia Campos Pavan Baptista, Michael West

**Affiliations:** I Universidade de São Paulo Escola de Enfermagem Departamento de Orientação Profissional São Paulo SP Brasil Universidade de São Paulo. Escola de Enfermagem. Departamento de Orientação Profissional. São Paulo, SP, Brasil; II Ecole de Management de Lyon EMLyon Lyon France Ecole de Management de Lyon, EMLyon. Lyon, France; III Universidad San Sebastián Facultad de Ciencias para el Cuidado de la Salud Santiago Chile Universidad San Sebastián. Facultad de Ciencias para el Cuidado de la Salud. Santiago, Región Metropolitana, Chile; IV Kamiyama Consultoria em Estatística Ltda São Paulo SP Brasil Kamiyama Consultoria em Estatística Ltda. São Paulo, SP, Brasil; V Universidade Federal do Recôncavo da Bahia Centro de Ciências da Saúde Santo Antônio de Jesus BA Brasil Universidade Federal do Recôncavo da Bahia. Centro de Ciências da Saúde. Santo Antônio de Jesus, BA, Brasil; VI Lancaster University Management School Department Organization Work and Technology Lancaster United Kingdom Lancaster University. Management School. Department Organization Work and Technology. Lancaster, United Kingdom

**Keywords:** Family Health Strategy, Workforce, organization & administration, Work Engagement, Job Satisfaction, Dental Health Services

## Abstract

**OBJECTIVE:**

To analyze the association between team climate, team characteristics and satisfaction at work in teams of the *Estratégia Saúde da Família com Saúde Bucal* (Family Health Strategy with Oral Health) (ESF with SB).

**METHODS:**

Cross-sectional correlational study with ESF teams with SB in the municipality of São Paulo. Universe of 1,328 teams and random sample of 124 teams with 1,231 professionals. Applied questionnaire with data teams’ characterization, team climate scale, and satisfaction at work. Analysis of validity, of climate and satisfaction scores through mean among professionals in each team, cluster analysis, association between variables by Pearson’s correlation and Chi-square, and tested linear regression model for the two factors of satisfaction at work.

**RESULTS:**

There was a directly proportional association between team climate and satisfaction at work. The better the climate with regard to team goals, the greater the intrinsic satisfaction at work and with the physical environment. The better the climate with regard to team goals and task orientation, the greater the satisfaction with hierarchical relations. The group with best team climate reported higher percentage of teams ranked with better satisfaction at work, and in the group with the worst team climate there was higher percentage of teams with the lowest satisfaction at work.

**CONCLUSIONS:**

The study provides consistent although moderate evidence of association between favorable teamwork climate and job satisfaction in ESF with SB. It emphasizes the dimensions of climate, common goals and task orientation, and may serve as subsidy for management and permanent education of teams, aiming at the quality of care to the health needs of users, family and community in APS.

## INTRODUCTION

The World Health Organization recommends health systems and their workforce to act following interprofessional collaboration and teamwork principles, to ensure the patient’s safety and quality health care^1^. *Atenção Primária à Saúde* (Primary Health Care) (APS), the gateway to the Brazilian National Health System (*Sistema Único de Saúde* SUS), still needs to consolidate teamwork to ensure universal access and quality of integral care^2^.

A study^3^ that summarizes evidence on teamwork showed its contribution toward reducing medical errors, and improving professional performance. It points out that no transformation of health care will be complete if it does not understand the characteristics of effective teams. It also suggests the need for studies on climate, and how it can affect teamwork^3^.

The present study examined teamwork based on the constructs of team climate and satisfaction at work. We used the concept of team climate perception shared by its members about policies, practices, and procedures adopted in health care^4^. A team is understood as a group of professionals who work together in a permanent or semi-permanent way (on-site or hybrid work), with regular interactions to perform their work^4^, which is characterized by common objectives, responsibilities and team identity, recognition of the role and work of the many areas, and interdependence of actions^5^.

Satisfaction at work is a construct that indicates the perception of both the professional and the team about the work experience. Edwin Locke defines satisfaction at work as a constructive or enjoyable state that leads to positive evaluation of the work by professionals regarding the content, possibilities of promotion, recognition, working conditions, and relationships with peers and leaders^6^.

The international literature suggests close relationship between team climate and satisfaction at work, the former being a relevant predictor of the latter^7^. A study conducted with hospital staff in Chile suggests that the influence of team climate on satisfaction at work is greater than the influence of leadership^7^. A study developed in the United States indicates that team climate is related to lower professional turnover, that the relationship between team climate and team constitution is partially mediated by satisfaction at work, as well as the relationship between team climate and lower burnout rates in teams with safe climate and focus on the quality of care^8^.

Team size and composition are a key element of teamwork^10,11^. Teams should be made up by professionals from areas with the required skills for health care, according to the patients’ profile. They should not exceed eight to twelve members, since large teams may face difficulties to interact, compete more for power, or even remove members from decision-making^11^. This was evidenced by a study on collaborative interprofessional practice in APS at the SUS, which showed an association between favorable teamwork climate, team size and length of time in the team^12^.

The organizational dimension also influences teamwork recognized as management tool. A survey highlighted that team leadership and management may help professionals to better deal with work difficulties, and produce better results for patients^3^. It approached team characteristics that express organizational dimensions such as team size, length of time being part of the team, and *Organizações Sociais* (Social Organizations) (OS) responsible for managing the *Unidade Básica de Saúde* (Basic Health Unit) (UBS) and teams. The OS are one of the managerial models provided for in the Brazilian health legal-institutional framework. These are private, nonprofit entities that perform public services through a management contract^13^. In the city of São Paulo, all UBSs are managed by OS that celebrate contracts with the *Secretaria Municipal de Saúde* (Municipal Health Secretariat) (SMS).

Literature highlights that effective teamwork should produce positive results for the population’s health, the patients’ experience, health professionals, and for the rational use of resources^14^. It also suggests the need for research on the effectiveness of teamwork.

In this research we reviewed teams from the *Estratégia Saúde da Família com Saúde Bucal* (ESF with SB). The ESF - a APS model implemented in the SUS since the 1990s - is pointed out in the literature as a model of excellence in APS^15^. It is structured based on teamwork and, as such, enables investigating the study phenomenon - teamwork in APS, in a context where it is consolidated. The choice to study ESF teams with SB is due to the recognition of the importance of diversity of knowledge and practices for effective teamwork^10,11^, that SB is part of integral care, and that after a decade of implementation of the *Brasil Sorridente* (Smiling Brazil) Program, it is necessary to expand the coverage and integration of SB in the ESF^16^.

The need to expand knowledge about the effectiveness of teamwork in the ESF for the professionals’ satisfaction at work is of great relevance, and contributes to the management and continuing education in APS.

The aim of this research is to analyze the association between team climate, team characteristics, and satisfaction at work among ESF teams with SB in the city of São Paulo.

## METHODS

Cross-sectional correlational study with teams of ESF with SB in UBSs in the city of São Paulo, which has approximately 12 million inhabitants distributed over six health regions.

In October 2020, there were in Brazil 43,456 ESF teams (63.87% coverage) and 29,176 SB teams (42.99%); in the city of São Paulo there were 1,448 ESF teams (40.77%) and 448 SB teams (13.74%)^17^.

The unit of analysis of the study is the ESF team with SB, and the research universe was defined based on the *Cadastro Nacional de Estabelecimentos de Saúde* (National Register of Health Establishments) - CNES, which in October 2017 showed 1,328 ESF teams with SB - a smaller number than the total number of teams listed in 2020 because not all of them included SB.

The inclusion criteria were: complete team with at least one professional of each category (community health agent, nursing assistant or technician, nurse, physician, oral health assistant or technician, dental surgeon), length of time working in the team: at least six months for the first four categories, and at least four months for the last two, which are *Saúde Bucal*.

After applying the inclusion criteria to the universe of 1,328 teams identified in the CNES, 174 ESF teams with SB were found, and formed the basis of the sample selected for this study.

A significance level of 5% was adopted with 87% power, which led to the calculation of a sample of 150 teams selected through simple random sampling with implicit stratification of length of time in the team of the youngest member.

Data were collected from January 3, 2018 to December 10, 2018, in the UBS by trained and supervised field researchers. The self-report questionnaire was applied in three parts: 1. Characteristics of teams: team size (number of members), length of time on the team (average length of time working as team member), those responsible for managing the UBS and teams (OS to which the UBS is linked), 2. Team climate scale, and 3. Satisfaction at work scale.

Team climate was measured using the Anderson’s and West’s team climate inventory^4^, applying the version validated in Portuguese^18^ with 38 items and four factors: participation in the team, with a composite reliability (CR) measure of 0.90 and 12 items (participation in decision-making, frequency of interactions, and information sharing); support for new ideas, with a CR of 0.95, and 8 items (approval and support for each member’s and team’s attempts to introduce new ideas to respond to health needs); team goals, with a CR of 0.95, and 11 items (clarity and individual and collective commitment to common goals); and task orientation with a CR of 0.95 and 11 items (individual and team responsibility with monitoring for the best quality of care).

We used the scale of satisfaction at work (S20/23), indicated in a review of the construct measurement instruments as the third most used in research^6^, validated in Portuguese with 20 items and three factors: satisfaction with hierarchical relations (α 0.92), satisfaction with the physical work environment (α 0.86), and intrinsic satisfaction at work (α 0.77), in a sample of 640 workers, 72.3% from the field of education and 27.7% from the health field^19^. The scales used are Likert-type (5 totally satisfied to 1 totally dissatisfied).

Validity evidence of both scales was evidenced by Exploratory structural equation modeling (ESEM) using the Mplus 7 software^20,21^. Internal consistency was assessed from the Composite Reliability coefficient [criterion CR > 0.70] (21)^22^.

The SPSS software version 20 was used to verify normality in data distribution via Kolmogorov-Smirnov test. Team climate and satisfaction at work were evaluated by mean scores of the professionals in each team, rescaled to range from 0 to 100. In order to identify homogeneous groups of teams, according to factors of team climate and satisfaction at work, we used the cluster analysis technique via the k-means partition method.

Associations between continuous variables were evaluated by Pearson’s correlation, and between categorical variables by the Chi-Square test. A multiple linear regression model was also tested for the two factors of satisfaction at work. A 5% significance level was used in the statistical tests. All analyses considered the structure of the sampling plan, that is, the sample expansion weights were incorporated into them. The analyses were performed using the Complex Samples module of SPSS version 20, which incorporates the information from the sampling plan.

The research was approved by the *Comitês de Ética em Pesquisa da Escola de Enfermagem* (Research Ethics Committees of the Nursing School) of USP (CAAE: 64385717.6.0000.5392) and of the *Secretaria Municipal de Saúde de São Paulo* (São Paulo Municipal Health Secretariat) (CAAE: 64385717.6.3001.0086), and all participants signed the Informed Consent Form.

## RESULTS

A sample of 124 teams composed of 1,231 ESF professionals with SB from five regions of the city was studied. There was a loss of 26 teams (17.3%) in relation to the estimated sample of 150, because of changes in the composition of teams due to dismissal, maternity leave, illness, etc.

The validation of the satisfaction at work scale with the study sample resulted in a new structure with two factors, different from the previous validation in Portuguese^18^. It kept the 20 items: factor 1 Intrinsic Satisfaction at Work and with the Physical Environment, with a CR of 0.91, and 12 items from the three subscales of the previous version; factor 2 Satisfaction with Hierarchical Relationships with a CR of 0.94, and the 8 items of the previous subscale.

The 124 ESF teams with SB studied were distributed in professional categories to better analyze their characteristics. From the total number of professionals included in the research, 42.2% are community health agents, 14.3% nursing assistants/technicians, 13.1% oral health assistants/technicians, 10.1% dental surgeons, 10.1% nurses, and 10.2% physicians/medical residents.

Variability was found both in the length of time working with the team (one to ten years), and in the team size (nine to fourteen members). We also found different mean scores of team climate (total and factors), and of satisfaction at work (total and factors), according to the OS that manages the UBS and teams. The teams reported climate means with statistically significant differences (p < 0.001), and a range of scores between 83.57 ± 1.25 and 72.87 ± 0.99, depending on the OS. The same occurred with the results of satisfaction at work (total) with statistically significant differences (p < 0.001), and range of scores between 82.19 ± 1.51 and 67.33 ± 0.73. (Table 1; Table 2).

**Table 1:** Characteristics of the teams (length of time on team, team size), team climate and satisfaction at work of the teams of ESF with SB in the municipality of São Paulo. São Paulo, 2019. (n = 124)

	Mean	95%CI	Standard Error	Minimum
Length of time working on the team (months)	61.83	59.91–63.74	0.97	14.70
Team size (registry)	11.41	11.29–11.54	0.06	9.00
Teamwork climate				
Participation in the team	78.77	78.14–79.40	0.32	50.69
Support to new ideas	75.03	74.34–75.72	0.35	51.04
Team objectives	74.87	74.31–75.43	0.28	49.16
Task orientation	76.31	75.56–77.06	0.38	47.35
Teamwork climate – total	76.21	75.62–76.81	0.30	49.47
Satisfaction at work				
Intrinsic satisfaction at work and with physical environment	75.24	74.44–76.03	0.40	47.73
Satisfaction with hierarchical relationships	74.78	73.92–75.64	0.43	47.81
Satisfaction at work – total	75.05	74.27–75.84	0.40	48.85

95%CI: 95% confidence interval.

**Table 2:** Mean and standard error of team climate scores (total and domains), and of satisfaction at work (total and domains) among ESF with SB teams by Social Organization. São Paulo, 2019. (n = 124).

	Social Organization																		p

	A (n = 10)		B (n = 6)		C (n = 37)		D (n = 17)		E (n = 18)		F (n = 17)		G (n = 2)		H (n = 3)		I (n = 14)		

	Number of teams of ESF with SB and percentage																		

	n	%	n	%	n	%	n	%	n	%	n	%	n	%	n	%	n	%	
	10	8.1	6	4.8	37	29.8	17	13.7	18	14.5	17	13.7	2	1.6	3	2.4	14	11.6	N/A
Teamwork climate																			
Teamwork climate - total	77.27 ± 0.76^b^		76.27 ± 0.60^b^		76.11 ± 0.52^b^		75.71 ± 0.70^b^		76.02 ± 0.86^b^		72.87 ± 0.99^C^		76.44 ± 0.14^b^		83.57 ± 1.25^a^		79.02 ± 0.77^b^		< 0.001
Participation in the team	79.22 ± 0.97^c^		77.60 ± 0.85^c^		78.09 ± 0.59^c^		78.43 ± 0.72^c^		78.49 ± 0.72^c^		76.18 ± 1.12^c^		81.92 ± 0.10^b^		86.45 ± 0.68^a^		82.56 ± 0.83^b^		< 0.001
Support to new ideas	75.26 ± 0.84^c^		73.60 ± 0.41^c^		74.55 ± 0.67^c^		74.69 ± 0.78^c^		75.62 ± 0.99 ^c^		71.13 ± 1.03^d^		78.4 ± 0.37^b^		84.10 ± 0.85^a^		78.69 ± 0.94^b^		< 0.001
Team objectives	76.37 ± 0.86^b^		74.46 ± 0.97^b^		75.43 ± 0.42^b^		74.74 ± 0.68^b^		74.17 ± 0.91^b^		72.06 ± 0.92^b^		70.34 ± 0.59^c^		80.95 ± 1.68^a^		76.30 ± 0.70^b^		< 0.001
Tasks orientation	78.01 ± 0.68^b^		79.60 ± 0.59^b^		76.09 ± 0.69^b^		74.93 ± 0.89^b^		76.43 ± 1.04^b^		71.68 ± 1.26^c^		78.25 ± 1.39^b^		84.00 ± 1.71^a^		79.50 ± 0.93^b^		< 0.001
Satisfaction at work																			
Total	77.31 ± 1.09^c^		76.72 ± 0.88^c^		74.67 ± 0.68^c^		73.48 ± 1.1^c^		74.40 ± 1.05^c^		72.92 ± 1.26^b^		67.33 ± 0.73^d^		82.19 ± 1.51^a^		78.64 ± 1.09^a^		< 0.001
Intrinsic and with physical environment	77.89 ± 1.05^a^		77.23 ± 0.78^a^		74.59 ± 0.72^a^		72.64 ± 1.14^b^		75.40 ± 1.03^a^		74.22 ± 1.24^a^		66.79 ± 0.67^c^		82.73 ± 2.34^a^		77.98 ± 1.10^a^		< 0.001
Hierarchical relationships	76.44 ± 1.36^b^		75.95 ± 1.19^b^		74.80 ± 0.73^b^		74.75 ± 1.12^b^		72.9 ± 1.18^c^		70.97 ± 1.42^c^		68.14 ± 0.83^c^		81.38 ± 0.32^a^		79.64 ± 1.19^a^		< 0.001

Mean ± standard error.

p: descriptive level in the general linear model considering the sampling plan.

^a, b, c, d^: Present different means according to multiple comparisons with Bonferroni’s correction.

The teams located in UBS managed by the social organizations H and I had the best averages of team climate and satisfaction at work (total and factors). The teams located in UBS managed by organizations F and G had, respectively, the lowest mean total team climate in the three factors, and the lowest mean satisfaction at work (total and factors). In turn, teams managed by the social organizations A, B and C reported similar averages in all the scores of team climate and satisfaction at work (Table 2).

In team climate, when comparing the four factors, the only one that showed significant differences among them was team participation, with the highest mean. The two factors of satisfaction at work factors showed no significant differences between them.

According to the results summarized in table 3, all the coefficients estimated to capture the correlation between team climate and satisfaction at work were significant, and indicated a moderate positive and directly proportional relationship between the dimensions analyzed.

**Table 3:** Association between team climate and satisfaction at work among ESF with SB teams in the municipality of São Paulo. São Paulo, 2019. (n = 124)

	Intrinsic satisfaction at work and with physical environment	Satisfaction with hierarchical relationships
Team climate – total	0.520^a^	0.619^a^
Participation in the team	0.420^a^	0.541^a^
Support to new ideas	0.465^a^	0.557^a^
Team objectives	0.531^a^	0.592^a^
Tasks orientation	0.492^a^	0.587^a^

^a^ p < 0.001.

Weak negative correlations were observed between team size and total team climate (r = -0.178; p = 0.048), and in three factors: Team participation factor (r = -0.191; p = 0.033), Support for new ideas (r = -0.205; p = 0.022), and Task orientation (r = -0.180; p = 0.046). This suggests that the larger the team, the lower the total team climate, and in the three subscales.

In order to better understand how teams behave in relation to the four climate factors and the two satisfaction factors, team typologies were generated. The analysis pointed to the configuration of four team climate groups: the CE1 group presented the highest means in the four factors; the CE2 group presented the second highest means in the four factors, and so on. The CE4 group comprised the teams with the lowest means in the four factors. The analysis of satisfaction at work indicated the configuration of five groups: the ST1 group had the highest means in the two factors; the ST2 group had the second highest means in the two factors; and, the ST5 group, the lowest means in the two factors.

As shown in Table 4, there was an association between the groups of satisfaction at work and work climate (p < 0.001). The group with the best team climate (CE1) had the highest percentage of teams classified as having the best satisfaction at work - ST1 compared to the other team climate groups. The CE3 and CE4 groups, with the lowest team climates, had no teams classified as ST1. The worst team climate group (CE4) had the highest percentage of teams with the lowest satisfaction at work.

**Table 4:** Association between groups of satisfaction at work and groups of ESF with SB team climate in the municipality of São Paulo. São Paulo, 2019.

	Team climate								Total		p

	CE1		CE2		CE3		CE4				
				
	n	%	n	%	n	%	n	%	n	%	
Satisfaction at work	**16**	**100,0**	**67**	**100.0**	**31**	**100.0**	**10**	**100.0**	**124**	**100.0**	< 0.001
ST1	9	56.3	10	14.9	0	0.0	0	0.0	19	15.3	
ST2	4	25.0	38	56.7	14	45.2	1	10.0	57	46.0	
ST3	1	6.3	9	13.4	2	6.5	2	20.0	14	11.3	
ST4	2	12.5	9	13.4	15	48.4	4	40.0	30	24.2	
ST5	0	0.0	1	1.5	0	0.0	3	30.0	4	3.2	

Note: Absolute and relative frequencies are weighted. The totaling of the subgroups may not coincide with the grand total due to weighting or lack of information.

p: descriptive level of the Chi-Square test considering the sampling plan.

The results of the linear regression with the factor of intrinsic satisfaction at work, and with physical environment as the dependent variable (Table 5), showed that the increased score of the team objective factor led to an increase in the satisfaction score. It was also observed that the longer the team has been together, the lower the intrinsic satisfaction at work.

**Table 5:** Multiple linear regression models for the two factors of job satisfaction of ESF teams with SB in the municipality of São Paulo. São Paulo, 2019. (n = 124)

	Dependent variable					

	Intrinsic satisfaction at work and with physical environment			Satisfaction with hierarchical relationships		
	
	Coefficient	95%CI	p	Coefficient	95%CI	p
Participation in the team	-0.05	-0.29 to 0.18	0,649	0,15	-0,07 to 0,38	0.185
Support to new ideas	0.12	-0.14 to 0.38	0,352	0,07	-0,14 to 0,28	0.503
Team objectives	0.48	0.26 to 0.70	< 0,001	0,44	0,24 to 0,64	< 0.001
Task orientation	0.17	-0.04 to 0.39	0,106	0,25	0,06 to 0,45	0.012
Team size	-0.07	-0.58 to 0.44	0,794	0,05	-0,45 to 0,55	0.855
Mean length of time (months)	-0.05	-0.09 to -0.01	0,012	-0,05	-0,1 to -0,01	0.018
Constant	9.77	-2.71 to 22.25	0,124	1,74	-11.7 to 15.18	0.798

Kolmogorov-Smirnov test: Intrinsic satisfaction at work and with physical environment (p = 0.656) and satisfaction with hierarchical relationships (p = 0,509).

Intrinsic satisfaction at work and with physical environment – R^2^ = 31,8%; Satisfaction with hierarchical relationships – R^2^ = 39,9%.

The second model tested with the satisfaction factor with hierarchical relations as the dependent variable, showed that the increase in scores of the factors team climate, team goals, and task orientation led to an increase in the score of satisfaction with hierarchical relations. Similar to intrinsic satisfaction at work and physical environment satisfaction, the longer the time on the team, the lower the satisfaction with hierarchical relations (Table 5).

The study showed that average length of time on the team and team climate (team goals) affected both satisfaction factors.

## DISCUSSION

The results showed greater intrinsic satisfaction at work and with the physical environment than with the hierarchical relations established management. A study carried out in APS also points out that the meaning that professionals assign to work increases satisfaction, and supervision produces dissatisfaction; the work environment (temperature and ventilation), unlike the present study, causes dissatisfaction^23^.

A correlation was observed between factors of team climate and satisfaction at work, as well as between the total score of climate and satisfaction, supporting the literature that indicates a relationship between the two constructs^7^.

Regarding team climate, we observed higher average for the factor team participation in relation to other factors, which refers to interactions among team members and security to give their opinion, and participate in decision-making. Effective, frequent and informal communication is a key element of teamwork^5,24^.

The association between team climate and satisfaction at work was also evidenced in the clustering results. The group with the most favorable climate for teamwork reported higher percentage of teams classified with higher satisfaction at work, and in the two groups with less favorable climate for teamwork there were no teams from the group with higher satisfaction scores. A multicentric study in Brazil, Portugal and Spain also showed the impact of climate on satisfaction at work^25^, which supports the literature that suggests team climate as an important predictor of satisfaction at work^7^.

Among the team climate factors, common goals and task orientation were the elements that most contributed to satisfaction at work, which suggests the importance of both dimensions for the implementation and performance of teams. The existence of common and clear objectives is described as a key characteristic of effective teams^11^ and is associated with the well-being of professionals and improvement in patient safety^26^.

It was also observed that the greater the team climate in relation to team goals and task orientation, the greater the satisfaction with hierarchical relationships. A research identified that ESF teams with higher scores in the dimension of task-orientation set aside more time in their routine for planning meetings and work evaluation, which can promote greater engagement and satisfaction at work^12^.

Satisfaction with hierarchical relationships is related to management and leadership of teams and health services, referring to collaborative management and leadership style, and its contribution to effective teamwork. An analysis of the role of leadership in the performance of interprofessional work showed its association with improved communication and innovation in teams, favoring job satisfaction^27^.

However, one must be cautious when interpreting the results that show an association between team climate and job satisfaction in ESF teams with SB, since a moderate correlation between the variables was verified, as well as great dispersion and small numbers in some crossings of the types of climate and satisfaction (clustering).

The study also showed an association between team characteristics, climate, and satisfaction. The longer the time on the team, the lower the intrinsic satisfaction at work and satisfaction with hierarchical relationships, as well as the total team climate. Research describes team stability as an incentive to shared work and joint decision-making, although length of time should not be too long to avoid excessive familiarity among team members^10,11^.

Team size has also affected the climate, showing that the larger it was, the lower the total team climate and of the three dimensions: team participation, support for new ideas, and task orientation. This result corroborates the analysis that points to team size, varying between eight to ten members, as a parameter of effective teams^11^. A study in APS of the SUS found an association between better team climate and shorter average length of time in the team (3 years better climate, and 4 years worse climate), as well as smaller average team size (7 professionals per team better climate, and 10 professionals per team worse climate)^12^.

The teams showed a profile similar to that of other ESF studies^28,29^, with variability in team length of time and size. Both point out to professional turnover, which generates instability in teams, and echoes on the bond with users, longitudinality, and trust among professionals^30^. Research has shown that the higher the job satisfaction, the lower the turnover of physicians in the Family Health Program in São Paulo^29^.

The study limitations include: the desired social response bias, which may affect the participants’ answers when using self-report instruments; the voluntary participation of teams, which may have produced selection bias; the absence of variables to characterize the OS responsible for managing the UBS and teams, which would allow analyzing OS-related variations among teams. Besides the characteristics of Social Organizations, further studies should investigate possible confounding variables that mediate the relationship between team climate and satisfaction at work, e.g., leadership style, organizational culture, etc.

Finally, the study provides consistent, albeit moderate, evidence of an association between team climate and satisfaction at work in the ESF with SB. This is particularly related to the presence of shared team goals and task orientation, which monitors the teams’ work toward achieving the best health care outcomes.

The results of the study are subsidy for management and continuing education of ESF teams with SB, as they show that effective teamwork with satisfaction of professionals requires support from managers, in particular in the definition of common goals, based on knowledge of the needs of users and community, and in the monitoring and reflection of teams on the care produced, seeking the best quality. These findings may also be applied to the management of the more than 43 thousand ESF teams in the country, and in other countries with health systems in which APS operates in an interprofessional manner.
