# Supplementary material for: Relationship between team climate and satisfaction at work in the Family Health Strategy
Source: Rev Saude Publica. 2021 Dec 16;55:117. doi: 10.11606/s1518-8787.2021055003307 (PMC8812825; doi:10.11606/s1518-8787.2021055003307)
Supplement: Translation

# Translation: Relações entre clima de equipe e satisfação no trabalho na Estratégia Saúde da Família

**Authors:** Marina Peduzzi, Heloise Lima Fernandes Agreli, Pilar Espinoza, Mitti Ayako Hara Koyama, Everson Meireles, Patrícia Campos Pavan Baptista, Michael West

**Affiliations:** I Brasil Universidade de São Paulo. Escola de Enfermagem. Departamento de Orientação Profissional. São Paulo, SP, Brasil; II France Ecole de Management de Lyon, EMLyon. Lyon, France; III Chile Universidad San Sebastián. Facultad de Ciencias para el Cuidado de la Salud. Santiago, Región Metropolitana, Chile; IV Brasil Kamiyama Consultoria em Estatística Ltda. São Paulo, SP, Brasil; V Brasil Universidade Federal do Recôncavo da Bahia. Centro de Ciências da Saúde. Santo Antônio de Jesus, BA, Brasil; VI United Kingdom Lancaster University. Management School. Department Organization Work and Technology. Lancaster, United Kingdom

**Keywords:** Estratégia Saúde da Família, Recursos Humanos, organização & administração, Engajamento no Trabalho, Satisfação no Emprego, Serviços de Saúde Bucal

## Abstract

**OBJETIVO:**

Analisar associação entre clima de equipe, características das equipes e satisfação no trabalho em equipes da Estratégia Saúde da Família com Saúde Bucal (ESF com SB).

**MÉTODOS:**

Correlacional transversal com equipes da ESF com SB no município de São Paulo. Universo de 1.328 equipes e amostra aleatória de 124 equipes com 1.231 profissionais. Aplicado questionário com dados de caracterização das equipes, escala de clima de equipe e de satisfação no trabalho. Realizada análise de validade, dos escores de clima e satisfação via média dos profissionais de cada equipe, análise de agrupamentos, associação entre variáveis pela correlação de Pearson e Qui-Quadrado e testado modelo de regressão linear para os dois fatores de satisfação no trabalho.

**RESULTADOS:**

Verificou-se associação diretamente proporcional entre clima de equipe e satisfação no trabalho. Quanto maior clima com relação a objetivos da equipe, maior satisfação intrínseca no trabalho e com ambiente físico, quanto maior clima com relação a objetivos da equipe e orientação para as tarefas, maior satisfação com as relações hierárquicas. Verificou-se no grupo com melhor clima de equipe, maior porcentagem de equipes classificadas com melhor satisfação no trabalho e no grupo de pior clima de equipe, maior porcentagem de equipes com a mais baixa satisfação no trabalho.

**CONCLUSÕES:**

O estudo traz evidências consistentes, embora moderadas, de associação entre clima favorável ao trabalho em equipe e satisfação no trabalho na ESF com SB, com destaque para as dimensões de clima, objetivos comuns e orientação para a tarefa, constituindo subsídio para a gestão e educação permanente das equipes, visando a qualidade da atenção às necessidades de saúde de usuários, família e comunidade na APS.

## INTRODUÇÃO

A Organização Mundial da Saúde recomenda que os sistemas de saúde e sua força de trabalho atuem orientados pela colaboração interprofissional e trabalho em equipe para segurança do paciente e qualidade da atenção à saúde^1^. A Atenção Primária à Saúde (APS), que constitui a porta de entrada do Sistema Único de Saúde (SUS) no Brasil, ainda precisa consolidar o trabalho em equipe para assegurar acesso universal e qualidade da atenção integral^2^.

Estudo^3^ que sintetiza evidências sobre trabalho em equipe demonstra sua contribuição para a redução de erros médicos e melhora no desempenho profissional. Aponta que nenhuma transformação dos cuidados de saúde será completa sem a compreensão das características de equipes efetivas e sugere a necessidade de estudos sobre o clima e como pode afetar o trabalho em equipe^3^.

Neste estudo investigou-se o trabalho em equipe com base nos construtos clima de equipe e satisfação no trabalho. Utilizou-se a concepção de clima de equipe como percepção compartilhada entre seus membros sobre políticas, práticas e procedimentos adotados na atenção à saúde^4^. Entende-se equipe como o conjunto de profissionais que atuam de forma presencial ou semipresencial, com interações regulares para realização do trabalho^4^, caracterizado pelo compartilhamento de objetivos, responsabilidades e identidade de equipe, reconhecimento do papel e do trabalho das diversas áreas e interdependência das ações^5^.

Satisfação no trabalho constitui construto que expressa a percepção do profissional e da equipe sobre a experiência de trabalho. Edwin Locke define a satisfação no trabalho como o estado construtivo ou de prazer que resulta na avaliação positiva do trabalho pelos profissionais em relação ao conteúdo, às possibilidades de promoção, ao reconhecimento, às condições de trabalho e às relações com colegas e superiores hierárquicos^6^.

A literatura internacional aponta estreita relação entre clima de equipe e satisfação no trabalho, sendo o primeiro um relevante preditor do segundo^7^. Pesquisa realizada com equipes hospitalares no Chile, sugere que a influência do clima de equipe sobre a satisfação no trabalho é maior do que a influência da liderança^7^. Estudo desenvolvido nos Estados Unidos, revela que o clima de equipe está relacionado a menor rotatividade de profissionais, que a relação entre clima e composição das equipes é parcialmente mediada pela satisfação no trabalho, assim como entre clima de equipe e menor índice de *burnout* em equipes com clima de segurança e foco na qualidade do cuidado^8^.

O tamanho da equipe e sua composição consiste elemento chave do trabalho em equipe^10,11^. Equipes precisam ser compostas por profissionais de áreas que reúnam competências necessárias aos cuidados à saúde, segundo o perfil dos pacientes e não devem exceder o número de oito a doze membros, uma vez que equipes grandes podem enfrentar dificuldades na interação, ter mais competição por poder ou mesmo afastamento de membros da tomada de decisão^11^, como demonstrou estudo sobre prática interprofissional colaborativa na APS do SUS, que comprovou associação entre clima favorável ao trabalho em equipe, tamanho da equipe e tempo na equipe^12^.

A dimensão organizacional também influencia o trabalho em equipe reconhecido como ferramenta de gestão. Uma pesquisa destacou que a liderança e a gestão de equipes podem ajudar os profissionais a lidarem melhor com as dificuldades do trabalho e a produzir melhores resultados para os pacientes^3^. Nesse sentido, foram estudadas características das equipes que expressam a dimensão organizacional, como tamanho da equipe, tempo na equipe e organização social (OS) responsável pela gestão da unidade básica de saúde (UBS) e equipes. As OS constituem um dos modelos de gestão que compõe o arcabouço jurídico-institucional brasileiro na área da saúde, e são entidades privadas, sem fins lucrativos, que executam serviços públicos por meio de contrato de gestão^13^. No município de São Paulo, todas as UBS são administradas por OS, por meio de contratos com a Secretaria Municipal de Saúde (SMS).

A literatura destaca que o trabalho em equipe efetivo deve produzir resultados positivos para a saúde da população, a experiência dos pacientes, os profissionais de saúde e para o uso racional dos recursos^14^, e coloca a necessidade de investigação sobre a efetividade do trabalho em equipe.

Nesta pesquisa estudou-se equipes da Estratégia Saúde da Família com Saúde Bucal (ESF com SB). A ESF, modelo de APS implantado no SUS desde os anos 1990, é apontada na literatura como modelo de excelência na APS^15^ e está estruturada com base no trabalho em equipe, o que propicia a investigação do fenômeno de estudo – trabalho em equipe na APS, em um contexto em que está consolidado. A opção por estudar equipes da ESF com SB deve-se ao reconhecimento da importância da diversidade de saberes e práticas para o trabalho em equipe efetivo^10,11^, que a SB faz parte da atenção integral e que, após uma década de implantação do Programa Brasil Sorridente, é necessário ampliar a cobertura e integração da SB na ESF^16^.

A necessidade de ampliação do conhecimento sobre a efetividade do trabalho em equipe na ESF para a satisfação no trabalho dos profissionais é de grande importância e contribui para a gestão e educação permanente na APS.

O objetivo da pesquisa é analisar a associação entre clima de equipe, características das equipes e satisfação no trabalho em equipes da ESF com SB na cidade de São Paulo.

## MÉTODOS

Estudo correlacional transversal, com equipes ESF com SB em UBS da cidade de São Paulo, que tem aproximadamente 12 milhões de habitantes distribuídos em seis regiões de saúde.

Em outubro de 2020, havia no Brasil 43.456 equipes de ESF (63,87% de cobertura) e 29.176 equipes de SB (42,99%), na cidade de São Paulo havia 1.448 equipes ESF (40,77%) e 448 equipes de SB (13,74%)^17^.

A unidade de análise do estudo é a equipe ESF com SB e o universo da pesquisa foi definido com base no Cadastro Nacional de Estabelecimentos de Saúde (CNES), que em outubro de 2017 apresentava 1.328 equipes ESF com SB, número menor que o total de equipes listadas em 2020 porque nem todas incluíam SB.

Os critérios de inclusão foram: equipe completa, com pelo menos um profissional de cada categoria (agente comunitário de saúde, auxiliar ou técnico de enfermagem, enfermeiro, médico, auxiliar ou técnico de saúde bucal, cirurgião dentista), tempo de atuação na equipe: há no mínimo seis meses para as primeiras quatro categorias e há no mínimo quatro meses para as duas últimas, que são Saúde Bucal.

Aplicados os critérios de inclusão no universo de 1.328 equipes identificadas no CNES, resultaram 174 equipes ESF com SB que constituíram a base da amostra selecionada para este estudo.

Foi adotado o nível de significante de 5% com um poder de 87%, o que levou a calcular uma amostra de 150 equipes, selecionadas via amostra aleatória simples com estratificação implícita de tempo de equipe do membro mais novo.

A coleta de dados foi realizada de 3 de janeiro de 2018 a 10 de dezembro de 2018, nas UBS, por pesquisadores de campo treinados e supervisionados. Aplicado questionário de autorrelato com três partes: 1. Características das equipes: tamanho da equipe (número de membros), tempo na equipe (média de tempo na equipe dos membros), os responsáveis pela gestão da UBS e equipes (OS a que está vinculada a UBS), 2. Escala de clima de equipe e 3. Escala de satisfação no trabalho.

O clima de equipe foi medido utilizando o inventario de clima de equipe de Anderson e West^4^, com aplicação da versão validada em português^18^ com 38 itens e quatro fatores: participação na equipe, com medida de confiabilidade composta (CC) de 0,90 e 12 itens (participação na tomada de decisão, frequência de interações e compartilhamento de informações); apoio para ideias novas, com CC de 0,95 e 8 itens (aprovação e apoio às tentativas de cada membro e da equipe de introduzir novas ideias para responder as necessidades de saúde); objetivos da equipe, com CC de 0,95 e 11 itens (clareza e comprometimento individual e coletivo com os objetivos comuns); e orientação para as tarefas com CC de 0,95 e 11 itens (responsabilidade individual e da equipe com monitoramento para a melhor qualidade do cuidado).

Foi utilizada a escala de satisfação no trabalho (S20/23), apontada em revisão de instrumentos de medida do construto como a terceira mais utilizada em pesquisas^6^, validada em português com 20 itens e três fatores: satisfação com relações hierárquicas (α 0,92), satisfação com ambiente físico de trabalho (α 0,86) e satisfação intrínseca no trabalho (α 0,77), em amostra de 640 trabalhadores, 72,3% do campo da educação e 27,7% da saúde^19^. As escalas utilizadas são do tipo Likert (5 totalmente satisfeito a 1 totalmente insatisfeito).

Evidências de validade das duas escalas foram demostradas por meio de Análises Fatoriais Confirmatórias e Exploratórias (ESEM) a partir do software Mplus 7^20,21^. A consistência interna foi avaliada a partir do coeficiente de Confiabilidade Composta [critério CC > 0,70] (21)^22^.

Foi utilizado o software SPSS versão 20 para verificar a normalidade na distribuição dos dados via teste de Kolmogorov-Smirnov. O clima de equipe e a satisfação no trabalho foram avaliados por médias de escores dos profissionais de cada equipe, reescalonados para que variassem de 0 a 100. Para identificar grupos homogêneos de equipes, de acordo com os fatores de clima de equipe e de satisfação no trabalho, utilizou-se a técnica de análise de agrupamentos via método da partição k-means.

Associações entre as variáveis contínuas foram avaliadas por correlação de Pearson e, entre variáveis categóricas, pelo teste Qui-Quadrado. Foi testado ainda um modelo de regressão linear múltipla para os dois fatores de satisfação no trabalho. Nos testes estatísticos foi utilizado o nível de significância de 5%. Para todas as análises foi considerada a estrutura do plano amostral, ou seja, foram incorporados os pesos de expansão da amostra. As análises foram realizadas utilizando-se o módulo *Complex Samples* do SPSS versão 20 que incorpora as informações do plano amostral.

A pesquisa foi aprovada pelos Comitês de Ética em Pesquisa da Escola de Enfermagem da USP (CAAE: 64385717.6.0000.5392) e da Secretaria Municipal de Saúde de São Paulo (CAAE: 64385717.6.3001.0086) e todos os participantes assinaram o Termo de Consentimento Livre e Esclarecido.

## RESULTADOS

Estudada amostra de 124 equipes compostas por 1.231 profissionais da ESF com SB de cinco regiões da cidade. Houve perda de 26 equipes (17,3%) em relação à amostra estimada de 150, por mudanças na composição das equipes devido a desligamento, afastamento por licença maternidade, adoecimento etc.

A validação da escala de satisfação no trabalho com a amostra do estudo resultou em nova estrutura com dois fatores, diferente da validação prévia em português^18^, mantendo os 20 itens: fator 1 Satisfação Intrínseca no Trabalho e com Ambiente Físico com CC de 0,91 e 12 itens provenientes das três subescalas da versão anterior; fator 2 Satisfação com Relações Hierárquica com CC de 0,94 e os 8 itens da subescala anterior.

As 124 equipes ESF com SB estudadas foram distribuídas em categorias profissionais para melhor analisar suas características. Do total de profissionais incluídos na pesquisa, 42,2% são agentes comunitários de saúde, 14,3% auxiliares/técnicos de enfermagem, 13,1% auxiliares/técnicos de saúde bucal, 10,1% cirurgiões dentistas, 10,1% enfermeiros e 10,2% médicos/médicos residentes.

Verificou-se variabilidade tanto no tempo de trabalho na equipe (um a dez anos), como no tamanho da equipe (de nove a catorze integrantes). Também constatou-se médias distintas de escores de clima de equipe (total e fatores) e de satisfação no trabalho (total e fatores), segundo a OS que faz a gestão da UBS e equipes. As equipes apresentaram médias de clima com diferenças estatisticamente significativas (p < 0,001) e faixa de escores entre 83,57 ± 1,25 e 72,87 ± 0,99, dependendo da OS. O mesmo ocorreu com os resultados de satisfação no trabalho (total) com diferenças estatisticamente significativas (p < 0,001) e faixa de escore entre 82,19 ± 1,51 e 67,33 ± 0,73. (Tabela 1; Tabela 2).

**Tabela 1:** Características das equipes (tempo na equipe, tamanho da equipe), clima de equipe e satisfação no trabalho das equipes de ESF com SB do município de São Paulo. São Paulo, 2019.

	Média	IC95%	Erro padrão	Mínimo
Tempo que trabalha na equipe (meses)	61,83	59,91–63,74	0,97	14,70
Tamanho da equipe (cadastro)	11,41	11,29–11,54	0,06	9,00
Clima do trabalho em equipe				
Participação na equipe	78,77	78,14–79,40	0,32	50,69
Apoio para ideias novas	75,03	74,34–75,72	0,35	51,04
Objetivos da equipe	74,87	74,31–75,43	0,28	49,16
Orientação para as tarefas	76,31	75,56–77,06	0,38	47,35
Clima do trabalho em equipe - total	76,21	75,62–76,81	0,30	49,47
Satisfação no Trabalho				
Satisfação intrínseca no trabalho e com ambiente físico	75,24	74,44–76,03	0,40	47,73
Satisfação com relações hierárquicas	74,78	73,92–75,64	0,43	47,81
Satisfação no trabalho – total	75,05	74,27–75,84	0,40	48,85

n = 124.

IC95%: intervalo de confiança de 95%.

**Tabela 2:** Média e Erro Padrão dos escores de clima de equipe (total e domínios) e de satisfação no trabalho (total e domínios) nas equipes ESF com SB por Organização Social. São Paulo. 2019.

	Organização social																		p

	A (n = 10)		B (n = 6)		C (n = 37)		D (n = 17)		E (n = 18)		F (n = 17)		G (n = 2)		H (n = 3)		I (n = 14)		

	Número de equipes ESF com SB e porcentagem																		

	n	%	n	%	n	%	n	%	n	%	n	%	n	%	n	%	n	%	
	10	8,1	6	4,8	37	29,8	17	13,7	18	14,5	17	13,7	2	1,6	3	2,4	14	11,6	N/A
Clima do trabalho em equipe																			
Clima do trabalho em equipe - total	77,27 ± 0,76^b^		76,27 ± 0,60^b^		76,11 ± 0,52^b^		75,71 ± 0,70^b^		76,02 ± 0,86^b^		72,87 ± 0,99^C^		76,44 ± 0,14^b^		83,57 ± 1,25^a^		79,02 ± 0,77^b^		< 0,001
Participação na equipe	79,22 ± 0,97^c^		77,60 ± 0,85^c^		78,09 ± 0,59^c^		78,43 ± 0,72^c^		78,49 ± 0,72^c^		76,18 ± 1,12^c^		81,92 ± 0,10^b^		86,45 ± 0,68^a^		82,56 ± 0,83^b^		< 0,001
Apoio para ideias novas	75,26 ± 0,84^c^		73,60 ± 0,41^c^		74,55 ± 0,67^c^		74,69 ± 0,78^c^		75,62 ± 0,99 ^c^		71,13 ± 1,03^d^		78,4 ± 0,37^b^		84,10 ± 0,85^a^		78,69 ± 0,94^b^		< 0,001
Objetivos da equipe	76,37 ± 0,86^b^		74,46 ± 0,97^b^		75,43 ± 0,42^b^		74,74 ± 0,68^b^		74,17 ± 0,91^b^		72,06 ± 0,92^b^		70,34 ± 0,59^c^		80,95 ± 1,68^a^		76,30 ± 0,70^b^		< 0,001
Orientação para as tarefas	78,01 ± 0,68^b^		79,60 ± 0,59^b^		76,09 ± 0,69^b^		74,93 ± 0,89^b^		76,43 ± 1,04^b^		71,68 ± 1,26^c^		78,25 ± 1,39^b^		84,00 ± 1,71^a^		79,50 ± 0,93^b^		< 0,001
Satisfação no trabalho																			
Total	77,31 ± 1,09^c^		76,72 ± 0,88^c^		74,67 ± 0,68^c^		73,48 ± 1,1^c^		74,40 ± 1,05^c^		72,92 ± 1,26^b^		67,33 ± 0,73^d^		82,19 ± 1,51^a^		78,64 ± 1,09^a^		< 0,001
Intrínseca e com ambiente físico	77,89 ± 1,05^a^		77,23 ± 0,78^a^		74,59 ± 0,72^a^		72,64 ± 1,14^b^		75,40 ± 1,03^a^		74,22 ± 1,24^a^		66,79 ± 0,67^c^		82,73 ± 2,34^a^		77,98 ± 1,10^a^		< 0,001
Relações hierárquicas	76,44 ± 1,36^b^		75,95 ± 1,19^b^		74,80 ± 0,73^b^		74,75 ± 1,12^b^		72,9 ± 1,18^c^		70,97 ± 1,42^c^		68,14 ± 0,83^c^		81,38 ± 0,32^a^		79,64 ± 1,19^a^		< 0,001

n = 124.

Média ± erro padrão.

p: nível descritivo do Modelo Linear Geral considerando-se o plano amostral.

^a, b, c, d^: Apresentam médias distintas segundo comparações múltiplas com correção de Bonferroni.

As equipes inseridas em UBS com gestão das organizações sociais H e I apresentaram melhores médias de clima de equipe e de satisfação no trabalho (total e fatores). As equipes localizadas em UBS com gestão das organizações F e G apresentaram, respectivamente, as menores médias de clima de equipe total, nos três fatores e as menores médias de satisfação no trabalho (total e fatores). Por sua vez, as equipes com gestão das organizações sociais A, B e C apresentaram médias similares em todos os escores de clima e de satisfação no trabalho (Tabela 2).

No clima de equipe, quando se comparam os quatro fatores, o único que mostrou diferenças significativas entre eles foi o de participação na equipe, com maior média. Os dois fatores de satisfação no trabalho não mostraram diferenças significativas entre eles.

Conforme resultados sumarizados na tabela 3, todos os coeficientes estimados para captar a correlação entre clima de equipe e satisfação no trabalho foram significativos e indicaram uma relação positiva moderada e diretamente proporcional entre as dimensões analisadas.

**Tabela 3:** Associação entre clima de equipe e satisfação no trabalho das equipes de ESF com SB do município de São Paulo. São Paulo, 2019.

	Satisfação intrínseca no trabalho e com ambiente físico	Satisfação com relações hierárquicas
Clima de equipe – total	0,520^a^	0,619^a^
Participação na equipe	0,420^a^	0,541^a^
Apoio para ideias novas	0,465^a^	0,557^a^
Objetivos da equipe	0,531^a^	0,592^a^
Orientação para as tarefas	0,492^a^	0,587^a^

n = 124.

^a^ p < 0,001.

Foram observadas correlações negativas fracas entre tamanho de equipe e clima de equipe total (r = -0,178; p = 0,048) e em três fatores: fator Participação na equipe (r = -0,191; p = 0,033), Apoio para ideias novas (r = -0,205; p = 0,022) e Orientação para as tarefas (r = -0,180; p = 0,046), apontando que quanto maior a equipe, menor o clima de equipe total e nas três subescalas.

Para se compreender melhor como as equipes se comportam em relação aos quatro fatores de clima e aos dois fatores de satisfação, foram geradas tipologias de equipes. A análise apontou a formação de quatro grupos de clima de equipe: o grupo CE1 apresentou as maiores médias nos quatros fatores; o grupo CE2 apresentou as segundas maiores médias nos quatros fatores e assim por diante. O grupo CE4 compreendeu as equipes com as menores médias nos quatro fatores. A análise da satisfação no trabalho apontou a formação de cinco grupos: o grupo ST1 apresentou as maiores médias nos dois fatores; o grupo ST2 apresentou as segundas maiores médias nos dois fatores e o ST5, as menores médias nos dois fatores.

Como apresenta a Tabela 4, verificou-se associação entre os grupos de satisfação no trabalho e de clima de trabalho (p < 0,001). No grupo de melhor clima de equipe (CE1), teve a maior porcentagem de equipes classificadas como tendo a melhor satisfação no trabalho – ST1, comparativamente aos demais grupos de clima de equipe. Os grupos CE3 e CE4, de menores climas de equipe, não apresentaram equipes classificadas do grupo ST1. O grupo de pior clima de equipe (CE4) apresentou a maior porcentagem de equipes com a mais baixa satisfação no trabalho.

**Tabela 4:** Associação entre os grupos de satisfação no trabalho e grupos de clima das equipes de ESF com SB do município de São Paulo. São Paulo. 2019.

	Clima de Equipe								Total		p

	CE1		CE2		CE3		CE4				
				
	n	%	n	%	n	%	n	%	n	%	
Satisfação no trabalho	**16**	**100,0**	**67**	**100,0**	**31**	**100,0**	**10**	**100,0**	**124**	**100,0**	< 0,001
ST1	9	56,3	10	14,9	0	0,0	0	0,0	19	15,3	
ST2	4	25,0	38	56,7	14	45,2	1	10,0	57	46,0	
ST3	1	6,3	9	13,4	2	6,5	2	20,0	14	11,3	
ST4	2	12,5	9	13,4	15	48,4	4	40,0	30	24,2	
ST5	0	0,0	1	1,5	0	0,0	3	30,0	4	3,2	

Frequências absoluta e relativa encontram-se ponderadas. A totalização dos subgrupos eventualmente poderá não coincidir com o total geral devido à ponderação ou a ausências de informações.

p: nível descritivo do teste de Qui-Quadrado considerando-se o plano amostral.

Os resultados da regressão linear com o fator satisfação intrínseca no trabalho e com ambiente físico como variável dependente (Tabela 5), mostrou que o aumento de escore do fator objetivo da equipe acarretou o aumento do escore de satisfação. Observou-se também que quanto maior o tempo de equipe, menor a satisfação intrínseca no trabalho.

**Tabela 5:** Modelos de regressão linear múltipla para os dois fatores de satisfação no trabalho das equipes de ESF com SB do município de São Paulo. São Paulo, 2019.

	Variável Dependente					

	Satisfação intrínseca no trabalho e com ambiente físico			Satisfação com relações hierárquicas		
	
	Coeficiente	IC95%	p	Coeficiente	IC95%	p
Participação na equipe	-0,05	-0,29 a 0,18	0,649	0,15	-0,07 a 0,38	0,185
Apoio para ideias novas	0,12	-0,14 a 0,38	0,352	0,07	-0,14 a 0,28	0,503
Objetivos da equipe	0,48	0,26 a 0,70	< 0,001	0,44	0,24 a 0,64	< 0,001
Orientação para as tarefas	0,17	-0,04 a 0,39	0,106	0,25	0,06 a 0,45	0,012
Tamanho da Equipe	-0,07	-0,58 a 0,44	0,794	0,05	-0,45 a 0,55	0,855
Tempo médio (meses)	-0,05	-0,09 a -0,01	0,012	-0,05	-0,1 a -0,01	0,018
Constante	9,77	-2,71 a 22,25	0,124	1,74	-11,7 a 15,18	0,798

n = 124.

Teste de Kolmogorov-Smirnov: Satisfação intrínseca no trabalho e com ambiente físico (p = 0,656) e Satisfação com relações hierárquicas (p = 0,509).

Satisfação intrínseca no trabalho e com ambiente físico – R^2^ = 31,8%; Satisfação com relações hierárquicas – R^2^ = 39,9%.

O segundo modelo testado com o fator satisfação com relações hierárquicas como variável dependente mostrou que o aumento de escores dos fatores do clima de equipe, objetivos da equipe e orientação para as tarefas acarreta o aumento no escore da satisfação com relações hierárquicas. De modo similar à satisfação intrínseca no trabalho e com ambiente físico, quanto maior o tempo de equipe, menor a satisfação com relações hierárquicas (Tabela 5).

O estudo mostrou que o tempo médio na equipe e o clima da equipe (objetivos da equipe) afetaram os dois fatores de satisfação.

## DISCUSSÃO

Os resultados mostraram maior satisfação intrínseca no trabalho e com ambiente físico do que com relações hierárquicas estabelecidas com a gestão. Estudo realizado na APS também aponta que o sentido atribuído ao trabalho pelos profissionais aumenta a satisfação e a supervisão produz insatisfação, o ambiente de trabalho (temperatura e ventilação), diferente da presente pesquisa, acarreta insatisfação^23^.

Foi verificada correlação entre os fatores de clima de equipe e satisfação no trabalho, assim como entre o escore total de clima e de satisfação, corroborando a literatura que aponta relação entre os dois construtos^7^.

Sobre o clima das equipes, observou-se maior média do fator participação na equipe em relação aos demais fatores, o que remete às interações entre os membros da equipe e à segurança em opinar e participar da tomada de decisão. A comunicação efetiva, frequente e informal constitui um elemento chave do trabalho em equipe^5,24^.

A associação entre o clima de equipe e a satisfação no trabalho também foi evidenciada nos resultados dos agrupamentos. No grupo com clima mais favorável ao trabalho em equipe, havia maior porcentagem de equipes classificadas com maior satisfação no trabalho e nos dois grupos com clima menos favorável ao trabalho em equipe, não havia equipes do grupo com escores mais altos de satisfação. Estudo multicêntrico nos contextos brasileiro, português e espanhol também evidenciou o impacto do clima na satisfação no trabalho^25^, o que corrobora a literatura que sugere clima de equipe como importante preditor de satisfação no trabalho^7^.

Entre os fatores de clima de equipe, os objetivos comuns e a orientação para as tarefas foram os elementos que mais contribuíram para satisfação no trabalho, o que sugere a importância das duas dimensões para a implantação e o desempenho das equipes. A existência de objetivos comuns e claros é descrita como característica-chave de equipes efetivas^11^ e está associada ao bem-estar dos profissionais e à melhoria na segurança do paciente^26^.

Observou-se também que quanto maior o clima de equipe em relação aos objetivos da equipe e à orientação para as tarefas, maior a satisfação com relações hierárquicas. Pesquisa identificou que equipes ESF com mais altos escores na dimensão orientação para tarefas, reservaram mais tempo de sua rotina para reuniões de planejamento e avaliação do trabalho, o que pode promover maior engajamento e satisfação no trabalho^12^.

A satisfação com as relações hierárquicas diz respeito à gestão e à liderança de equipes e de serviços de saúde, remetendo ao estilo de gestão e liderança colaborativa e sua contribuição para o efetivo trabalho em equipe. Análise do papel da liderança no desempenho do trabalho interprofissional mostrou sua associação à melhora da comunicação e inovação nas equipes, favorecendo a satisfação no trabalho^27^.

Contudo, é preciso ter cautela na interpretação dos resultados que mostram associação entre o clima de equipe e a satisfação no trabalho nas equipes de ESF com SB, visto que foi verificada correlação moderada entre as variáveis, bem como grande dispersão e números pequenos em alguns cruzamentos dos tipos de clima e satisfação (agrupamentos).

O estudo também mostrou associação entre características da equipe, clima e satisfação. Quanto maior o tempo na equipe, menor a satisfação intrínseca no trabalho e a satisfação com as relações hierárquicas, bem como o clima de equipe total. Pesquisas descrevem a estabilidade da equipe como estímulo ao trabalho compartilhado e à tomada de decisão conjunta, embora o tempo não deva ser muito extenso para evitar excessiva familiaridade entre membros da equipe^10,11^.

O tamanho da equipe também afetou o clima mostrando que quanto maior, menor o clima de equipe total e das três dimensões: participação na equipe, apoio para novas ideias e orientação para as tarefas. Este resultado corrobora análise que aponta o tamanho da equipe, variando entre oito e dez membros, como parâmetro de equipes efetivas^11^. Pesquisa na APS do SUS verificou associação entre melhor clima de equipe e menor média de tempo da equipe (3 anos melhor clima e 4 anos pior clima), bem como menor média de tamanho da equipe (7 profissionais por equipe melhor clima e 10 profissionais por equipe pior clima)^12^.

As equipes mostraram perfil semelhante ao de outros estudos da ESF^28,29^, com variabilidade do tempo e do tamanho da equipe, ambos apontando para rotatividade dos profissionais, o que gera instabilidade das equipes e têm repercussões no vínculo com os usuários, na longitudinalidade e na confiança entre os profissionais^30^. Pesquisa demonstrou que quanto maior a satisfação no trabalho, menor a rotatividade dos médicos do Programa Saúde da Família em São Paulo^29^.

Consideram-se como limitações do estudo: o viés de resposta social desejada, que pode afetar as respostas dos participantes na utilização de instrumentos de auto relato; a participação voluntária das equipes, que pode ter produzido viés de seleção; a ausência de variáveis de caracterização da OS responsável pela gestão da UBS e equipes, que permitiriam analisar as variações entre equipes decorrentes da OS. Além das características das organizações sociais, estudos futuros deveriam indagar possíveis variáveis de confusão que mediam a relação entre clima de equipe e satisfação no trabalho, por exemplo, estilo de liderança, cultura organizacional etc.

Por fim, o estudo traz evidências consistentes, embora moderadas, de associação entre clima de equipe e satisfação no trabalho na ESF com SB. Isso está relacionado, em particular, à presença de objetivos comuns de equipe e de orientação para as tarefas. Esta se refere ao monitoramento que profissionais e equipe realizam para alcançar os melhores resultados na atenção à saúde.

Os resultados do estudo constituem subsídio para a gestão e a educação permanente das equipes ESF com SB, pois evidenciam que o trabalho em equipe efetivo com satisfação dos profissionais requer apoio dos gerentes, em particular, na definição de objetivos comuns, com base no conhecimento das necessidades de usuários e comunidade, e no monitoramento e reflexão das equipes sobre o cuidado produzido, buscando a melhor qualidade. Esses achados também podem ser aplicados à gestão das mais de 43 mil equipes de ESF do país, e em outros países com sistemas de saúde em que a APS atue de forna interprofissional.
